# 4-(3-Eth­oxy-4-hydroxy­styr­yl)-1-methyl­pyridinium tosyl­ate monohydrate

**DOI:** 10.1107/S1600536808041007

**Published:** 2008-12-10

**Authors:** S. Murugavel, A. SubbiahPandi, C. Srikanth, S. Kalainathan

**Affiliations:** a Department of Physics, Thanthai Periyar Government Institute of Technology, Vellore 632 002, India; b Department of Physics, Presidency College (Autonomous), Chennai 600 005, India; c School of Science & Humanities, VIT University, Vellore 632 014, India

## Abstract

In the title compound, C_16_H_18_NO_2_^+^·C_7_H_7_O_3_S^−^·H_2_O, the dihedral angle between the pyridyl and benzene rings of the pyridinium cation is 0.2 (1)°. The benzene ring of the tosyl­ate anion makes a dihedral angle of 4.8 (2)° with the best mean plane of the pyridinium cation. The pyridinium cation and the tosyl­ate anion are hydrogen bonded to the water mol­ecule, and the crystal packing is further stabilized by inter­molecular C—H⋯O and π–π inter­actions [centroid–centroid separations of 3.648 (3) and 3.594 (2) Å.

## Related literature

For a related structure, see: Zhang *et al.* (1997 ▶). For mol­ecular compounds with non-linear optical properties, see: Bosshard *et al.* (1995 ▶); Nalwa & Miyata (1997 ▶); Lee & Kim (1999 ▶).
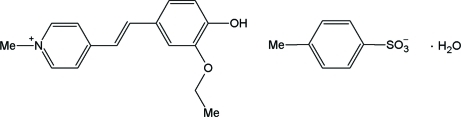


## Experimental

### 

#### Crystal data

C_16_H_18_NO_2_^+^·C_7_H_7_O_3_S^−^·H_2_O
*M* *_r_* = 445.52
Monoclinic, 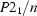

*a* = 13.7700 (4) Å
*b* = 9.7125 (2) Å
*c* = 17.3394 (5) Å
β = 104.059 (2)°
*V* = 2249.53 (10) Å^3^
*Z* = 4
Mo *K*α radiation
μ = 0.18 mm^−1^
*T* = 293 (2) K
0.25 × 0.17 × 0.16 mm

#### Data collection

Bruker APEXII CCD area-detector diffractometer
Absorption correction: multi-scan (*SADABS*; Sheldrick, 1996 ▶) *T*_min_ = 0.961, *T*_max_ = 0.975
48719 measured reflections
5310 independent reflections
3610 reflections with *I* > 2σ(*I*)
*R*_int_ = 0.034

#### Refinement

*R*[*F* ^2^ > 2σ(*F* ^2^)] = 0.057
*wR*(*F* ^2^) = 0.187
*S* = 1.03
5310 reflections
290 parameters
3 restraints
H atoms treated by a mixture of independent and constrained refinement
Δρ_max_ = 0.72 e Å^−3^
Δρ_min_ = −0.30 e Å^−3^

### 

Data collection: *APEX2* (Bruker, 2004 ▶); cell refinement: *APEX2* and *SAINT* (Bruker, 2004 ▶); data reduction: *SAINT* and *XPREP* (Bruker, 2004 ▶); program(s) used to solve structure: *SHELXS97* (Sheldrick, 2008 ▶); program(s) used to refine structure: *SHELXL97* (Sheldrick, 2008 ▶); molecular graphics: *ORTEP-3* (Farrugia, 1997 ▶); software used to prepare material for publication: *SHELXL97* and *PLATON* (Spek, 2003 ▶).

## Supplementary Material

Crystal structure: contains datablocks global, I. DOI: 10.1107/S1600536808041007/lx2079sup1.cif

Structure factors: contains datablocks I. DOI: 10.1107/S1600536808041007/lx2079Isup2.hkl

Additional supplementary materials:  crystallographic information; 3D view; checkCIF report

## Figures and Tables

**Table 1:** Hydrogen-bond geometry (Å, °)

*D*—H⋯*A*	*D*—H	H⋯*A*	*D*⋯*A*	*D*—H⋯*A*
O1—H1*O*⋯O6^i^	0.82	1.82	2.632 (3)	170
O6—H6*OB*⋯O3	0.85 (1)	2.36 (2)	2.692 (3)	103.9 (19)
O6—H6*OA*⋯O5^ii^	0.86 (3)	1.98 (3)	2.832 (4)	169 (3)
C4—H4⋯O4	0.93	2.53	3.426 (3)	161
C5—H5⋯O1^iii^	0.93	2.59	3.217 (3)	125

Symmetry codes: (i) 
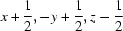
; (ii) 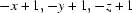; (iii) 
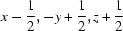
.

**Fig. 1.**
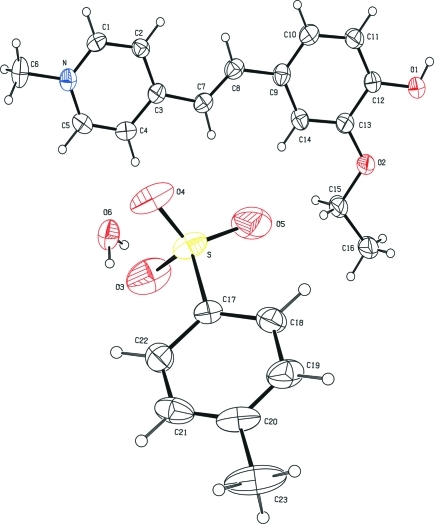
The molecular structure of title compound showing 30% probability displacement ellipsoids.

**Fig. 2.**
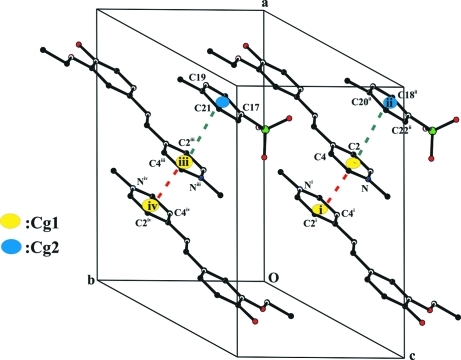
2. π—π interactions (dotted lines) in the title compound. Cg denotes ring centroid. [Symmetry code: (i) -x+1, -y, -z+1; (ii) x, y-1, z; (iii) x, y+1, z; (iv) -x+1, -y+1, -z+1.]

## supplementary crystallographic information

### Comment

The synthesis and study of molecular compounds with non linear optical (NLO) properties has attracted much attention, because such materials hold promise for applications in optoelectronic and photonic devices (Bosshard *et al.*,  1995; Nalwa & Miyata, 1997). In order to create efficient quadratic (second–order) NLO materials, both the molecular and bulk properties must be optimized. Within the diverse range of existing NLO compounds, styrylpyridinium salts are particularly attractive for device applications (Lee & Kim, 1999). Against this background, and in order to obtain detailed information on molecular conformations in the solid state, X-ray studies of the title compounds (I) have been carried out.

X-Ray analysis confirms the molecular structure and atom connectivity for (I), as illustrated in Fig. 1. The dihedral angle between the pyridyl and phenyl rings of the pyridinium cation is 0.2 (1)°.  The benzene ring of the tosylate anion makes a dihedral angle of 4.8 (2)° with the best mean plane of the pyridinium cation.  The bond lengths N1–C7, C13–O25 and C14–O26 are normal and comparable with the corresponding values observed in the related structure. (Zhang *et al.*, 1997)

The presence of water molecules in the crystal structure of (I) leads to a three dimensional network of hydrogen bonds invoving water, the tosylate anion and the pyridinium cation (Table 1). In addition, the crystal packing is further stabilized by intermolecular C—H···O (Table.1) and π—π interactions with a Cg1···Cg1^i^  and a Cg1—Cg2^ii^ separation of 3.648 (3) Å and 3.594 Å, respectively (Fig. 2; Cg1 and Cg2 are the centroids of the N/C1-C5 pyridine ring and C17-C22 benzene ring, respectively, symmetry code as in Fig. 2).

### Experimental

HEST (4-[2-(4-hydroxy-3-ethoxyphenyl) ethenyl]-1-methylpyridinium 4-tolylsulfonate hydrate ) was synthesized by the condensation of 4-methyl N-methyl pyridinum Tosylate, which is prepared from 4-Picoline (Merck, 99%) , methyl toluene sulphonate (Merck, 98%) and 4-hydroxy-3-ethoxy-Benzaldehyde (High Media, 98%) in the presence of piperidine as catalyst. The step by step synthesis procedure of HEST is as follows: Picoline (10.31 ml, 0.105 mol %) and methyl toluene sulphonate (15.88 ml, 0.105 mol %) is added into toluene (200ml) (Merck, 98%) is taken in a round bottom flask (500 ml) of Dean-stark apparatus. This mixture is heated until formation of white salt, which is insoluble in toluene. While boiling Di-methyl formamide (DMF) (Merck, 98%) is added until the white salt are dissolved. Now 4-hydroxy-3-ethoxy-Benzaldehyde (0.105 mol %) is added. Few drops of Piperidine also added as catalyst. The mixture is then refluxed with Dean-stark trap to remove water. After more than equivalent amount of water is collected, the reactants are cooled to room temperature and synthesized orange color HEST is collected. To prevent the absorption of water from the atmosphere, the synthesized material is placed in the oven at 100°C for 1 hour. Purified single crystals suitable for X-ray diffraction was obtained by successive recrystallization process of a methonal solution.

### Refinement

H atoms of the water were located in a difference fourier map, and were refined with distance restraints of O—H = 0.85(0.01) Å and H···H = 1.25(0.01) Å and all other H atoms were fixed geometrically and allowed to ride on their parent atoms, with  C—H = 0.93–0.98 Å with *U*_iso_(H)= 1.5*U*_eq_ (methyl H) and 1.2*U*_eq_ (for other H atoms).

### Crystal data

**Table:**

C_16_H_18_NO_2_^+^·C_7_H_7_O_3_S^−^·H_2_O	*F*(000) = 944
*M**_r_* = 445.52	*D*_x_ = 1.315 Mg m^−^^3^
Monoclinic, *P*2_1_/*n*	Mo *K*α radiation, λ = 0.71073 Å
Hall symbol: -P 2yn	Cell parameters from 5318 reflections
*a* = 13.7700 (4) Å	θ = 2.2–27.8°
*b* = 9.7125 (2) Å	µ = 0.18 mm^−^^1^
*c* = 17.3394 (5) Å	*T* = 293 K
β = 104.059 (2)°	Block, orange
*V* = 2249.53 (10) Å^3^	0.25 × 0.17 × 0.16 mm
*Z* = 4	

### Data collection

**Table:**

Bruker APEXII CCD area-detector diffractometer	5310 independent reflections
Radiation source: fine-focus sealed tube	3610 reflections with *I* > 2σ(*I*)
graphite	*R*_int_ = 0.034
Detector resolution: 10 pixels mm^-1^	θ_max_ = 27.8°, θ_min_ = 2.2°
ω scans	*h* = −18→18
Absorption correction: multi-scan (*SADABS*; Sheldrick, 1996)	*k* = −12→12
*T*_min_ = 0.961, *T*_max_ = 0.975	*l* = −22→22
48719 measured reflections	

### Refinement

**Table:**

Refinement on *F*^2^	Primary atom site location: structure-invariant direct methods
Least-squares matrix: full	Secondary atom site location: difference Fourier map
*R*[*F*^2^ > 2σ(*F*^2^)] = 0.057	Hydrogen site location: difference Fourier map
*wR*(*F*^2^) = 0.187	H atoms treated by a mixture of independent and constrained refinement
*S* = 1.03	*w* = 1/[σ^2^(*F*_o_^2^) + (0.0893*P*)^2^ + 1.1253*P*] where *P* = (*F*_o_^2^ + 2*F*_c_^2^)/3
5310 reflections	(Δ/σ)_max_ < 0.001
290 parameters	Δρ_max_ = 0.72 e Å^−^^3^
3 restraints	Δρ_min_ = −0.30 e Å^−^^3^

### Special details

**Table:**

Geometry. All esds (except the esd in the dihedral angle between two l.s. planes) are estimated using the full covariance matrix. The cell esds are taken into account individually in the estimation of esds in distances, angles and torsion angles; correlations between esds in cell parameters are only used when they are defined by crystal symmetry. An approximate (isotropic) treatment of cell esds is used for estimating esds involving l.s. planes.
Refinement. Refinement of F^2^ against ALL reflections. The weighted R-factor wR and goodness of fit S are based on F^2^, conventional R-factors R are based on F, with F set to zero for negative F^2^. The threshold expression of F^2^ > 2sigma(F^2^) is used only for calculating R-factors(gt) etc. and is not relevant to the choice of reflections for refinement. R-factors based on F^2^ are statistically about twice as large as those based on F, and R- factors based on ALL data will be even larger.

### Fractional atomic coordinates and isotropic or equivalent isotropic displacement parameters (Å^2^)

**Table:**

	*x*	*y*	*z*	*U*_iso_*/*U*_eq_	
S	0.72581 (5)	0.48936 (6)	0.59449 (4)	0.0646 (2)	
O1	0.92457 (12)	0.27591 (17)	0.20090 (10)	0.0589 (4)	
H1O	0.9248	0.2213	0.1649	0.088*	
O2	0.91242 (13)	0.42417 (17)	0.32358 (10)	0.0604 (4)	
O3	0.62950 (16)	0.5133 (2)	0.61455 (17)	0.0998 (8)	
O4	0.78329 (17)	0.3912 (2)	0.64784 (16)	0.0958 (8)	
O5	0.7116 (2)	0.4619 (2)	0.51204 (15)	0.1024 (8)	
O6	0.44858 (19)	0.3921 (3)	0.58789 (15)	0.0855 (6)	
H6OA	0.399 (2)	0.427 (3)	0.5530 (19)	0.16 (2)*	
H6OB	0.4608 (18)	0.4682 (16)	0.6128 (13)	0.050 (7)*	
N	0.55785 (13)	−0.0896 (2)	0.63677 (11)	0.0514 (5)	
C1	0.56915 (16)	−0.1585 (2)	0.57240 (14)	0.0515 (5)	
H1	0.5445	−0.2477	0.5633	0.062*	
C2	0.61625 (16)	−0.0993 (2)	0.52038 (13)	0.0484 (5)	
H2	0.6230	−0.1481	0.4758	0.058*	
C3	0.65447 (15)	0.0336 (2)	0.53320 (13)	0.0477 (5)	
C4	0.64007 (18)	0.1008 (2)	0.60023 (15)	0.0565 (6)	
H4	0.6637	0.1902	0.6110	0.068*	
C5	0.59222 (18)	0.0384 (3)	0.65010 (15)	0.0571 (6)	
H5	0.5832	0.0856	0.6945	0.069*	
C6	0.5107 (2)	−0.1567 (4)	0.69445 (17)	0.0814 (9)	
H6A	0.5615	−0.1870	0.7395	0.122*	
H6B	0.4723	−0.2345	0.6699	0.122*	
H6C	0.4673	−0.0924	0.7116	0.122*	
C7	0.70733 (17)	0.1044 (2)	0.48149 (14)	0.0547 (6)	
H7	0.7258	0.1957	0.4928	0.066*	
C8	0.73041 (18)	0.0465 (3)	0.41971 (15)	0.0578 (6)	
H8	0.7107	−0.0447	0.4098	0.069*	
C9	0.78346 (17)	0.1085 (3)	0.36470 (14)	0.0545 (6)	
C10	0.79432 (19)	0.0317 (3)	0.30061 (16)	0.0616 (6)	
H10	0.7700	−0.0580	0.2944	0.074*	
C11	0.84097 (18)	0.0864 (3)	0.24558 (15)	0.0581 (6)	
H11	0.8466	0.0335	0.2022	0.070*	
C12	0.87921 (16)	0.2176 (2)	0.25382 (13)	0.0486 (5)	
C13	0.87119 (17)	0.2968 (2)	0.31965 (13)	0.0503 (5)	
C14	0.82272 (17)	0.2423 (3)	0.37394 (13)	0.0541 (5)	
H14	0.8161	0.2952	0.4171	0.065*	
C15	0.9068 (2)	0.5061 (3)	0.39048 (17)	0.0719 (8)	
H15A	0.9420	0.4613	0.4393	0.086*	
H15B	0.8376	0.5191	0.3922	0.086*	
C16	0.9543 (3)	0.6418 (4)	0.3816 (2)	0.1043 (13)	
H16A	0.9518	0.6998	0.4259	0.156*	
H16B	0.9189	0.6851	0.3332	0.156*	
H16C	1.0228	0.6276	0.3801	0.156*	
C17	0.78797 (16)	0.6487 (2)	0.61343 (14)	0.0476 (5)	
C18	0.8387 (2)	0.7012 (3)	0.56113 (17)	0.0652 (7)	
H18	0.8428	0.6513	0.5163	0.078*	
C19	0.8839 (2)	0.8288 (3)	0.5755 (2)	0.0784 (9)	
H19	0.9184	0.8637	0.5398	0.094*	
C20	0.8792 (2)	0.9043 (3)	0.6399 (2)	0.0763 (9)	
C21	0.8292 (2)	0.8499 (3)	0.69306 (18)	0.0749 (9)	
H21	0.8265	0.8996	0.7383	0.090*	
C22	0.78351 (19)	0.7234 (3)	0.67996 (14)	0.0592 (6)	
H22	0.7496	0.6884	0.7160	0.071*	
C23	0.9251 (3)	1.0473 (3)	0.6528 (3)	0.133 (2)	
H23A	0.9759	1.0560	0.6237	0.200*	
H23B	0.9543	1.0611	0.7084	0.200*	
H23C	0.8740	1.1151	0.6343	0.200*	

### Atomic displacement parameters (Å^2^)

**Table:**

	*U*^11^	*U*^22^	*U*^33^	*U*^12^	*U*^13^	*U*^23^
S	0.0666 (4)	0.0422 (3)	0.0802 (5)	−0.0071 (3)	0.0084 (3)	0.0070 (3)
O1	0.0698 (10)	0.0593 (9)	0.0532 (9)	−0.0040 (8)	0.0257 (8)	−0.0002 (7)
O2	0.0762 (11)	0.0561 (9)	0.0528 (9)	−0.0154 (8)	0.0230 (8)	−0.0076 (7)
O3	0.0602 (12)	0.0806 (14)	0.156 (2)	−0.0146 (10)	0.0220 (13)	0.0169 (14)
O4	0.0885 (14)	0.0532 (11)	0.133 (2)	0.0007 (10)	0.0020 (13)	0.0301 (12)
O5	0.140 (2)	0.0646 (12)	0.0956 (17)	−0.0186 (13)	0.0156 (15)	−0.0220 (12)
O6	0.0890 (16)	0.0924 (16)	0.0747 (14)	−0.0218 (13)	0.0189 (12)	0.0171 (13)
N	0.0447 (10)	0.0609 (11)	0.0485 (11)	0.0068 (8)	0.0112 (8)	0.0105 (9)
C1	0.0492 (12)	0.0417 (10)	0.0614 (14)	0.0022 (9)	0.0090 (10)	0.0038 (10)
C2	0.0493 (11)	0.0485 (11)	0.0476 (12)	0.0041 (9)	0.0124 (9)	−0.0017 (9)
C3	0.0425 (11)	0.0489 (11)	0.0498 (12)	0.0038 (9)	0.0074 (9)	0.0055 (9)
C4	0.0575 (13)	0.0452 (11)	0.0642 (15)	0.0007 (10)	0.0101 (11)	−0.0056 (10)
C5	0.0561 (13)	0.0622 (14)	0.0523 (13)	0.0067 (11)	0.0121 (11)	−0.0102 (11)
C6	0.0753 (18)	0.109 (2)	0.0651 (18)	0.0012 (17)	0.0274 (14)	0.0299 (16)
C7	0.0548 (13)	0.0484 (11)	0.0597 (14)	−0.0057 (10)	0.0116 (11)	0.0003 (10)
C8	0.0580 (14)	0.0521 (12)	0.0621 (15)	−0.0083 (10)	0.0120 (11)	−0.0001 (11)
C9	0.0495 (12)	0.0610 (13)	0.0528 (13)	−0.0070 (10)	0.0125 (10)	0.0046 (10)
C10	0.0634 (15)	0.0551 (13)	0.0673 (16)	−0.0108 (11)	0.0179 (12)	−0.0025 (11)
C11	0.0614 (14)	0.0585 (13)	0.0563 (14)	−0.0046 (11)	0.0181 (11)	−0.0078 (11)
C12	0.0448 (11)	0.0556 (12)	0.0451 (12)	0.0015 (9)	0.0105 (9)	0.0028 (9)
C13	0.0499 (12)	0.0525 (12)	0.0463 (12)	−0.0036 (9)	0.0072 (9)	−0.0002 (9)
C14	0.0550 (13)	0.0624 (13)	0.0453 (12)	−0.0024 (10)	0.0131 (10)	−0.0032 (10)
C15	0.094 (2)	0.0704 (16)	0.0586 (15)	−0.0280 (14)	0.0324 (14)	−0.0164 (12)
C16	0.163 (3)	0.077 (2)	0.098 (2)	−0.052 (2)	0.079 (2)	−0.0346 (18)
C17	0.0463 (11)	0.0381 (10)	0.0570 (13)	0.0030 (8)	0.0101 (9)	0.0011 (9)
C18	0.0755 (16)	0.0488 (12)	0.0830 (18)	−0.0006 (11)	0.0420 (14)	−0.0084 (12)
C19	0.0653 (16)	0.0535 (14)	0.127 (3)	−0.0044 (12)	0.0430 (17)	0.0067 (16)
C20	0.0515 (14)	0.0437 (12)	0.119 (3)	0.0022 (11)	−0.0074 (15)	−0.0091 (15)
C21	0.0733 (17)	0.0632 (15)	0.0715 (18)	0.0183 (14)	−0.0146 (14)	−0.0236 (14)
C22	0.0603 (14)	0.0656 (14)	0.0489 (13)	0.0136 (11)	0.0076 (11)	0.0035 (11)
C23	0.083 (2)	0.0527 (17)	0.234 (6)	−0.0123 (16)	−0.021 (3)	−0.023 (3)

### Geometric parameters (Å, °)

**Table:**

S—O5	1.420 (3)	C9—C10	1.377 (4)
S—O4	1.427 (2)	C9—C14	1.401 (3)
S—O3	1.469 (2)	C10—C11	1.380 (3)
S—C17	1.760 (2)	C10—H10	0.9300
O1—C12	1.353 (3)	C11—C12	1.373 (3)
O1—H1O	0.8200	C11—H11	0.9300
O2—C13	1.356 (3)	C12—C13	1.403 (3)
O2—C15	1.424 (3)	C13—C14	1.385 (3)
O6—H6OA	0.86 (3)	C14—H14	0.9300
O6—H6OB	0.85 (1)	C15—C16	1.496 (4)
N—C5	1.330 (3)	C15—H15A	0.9700
N—C1	1.342 (3)	C15—H15B	0.9700
N—C6	1.471 (3)	C16—H16A	0.9600
C1—C2	1.360 (3)	C16—H16B	0.9600
C1—H1	0.9300	C16—H16C	0.9600
C2—C3	1.391 (3)	C17—C18	1.371 (3)
C2—H2	0.9300	C17—C22	1.377 (3)
C3—C4	1.389 (3)	C18—C19	1.382 (4)
C3—C7	1.457 (3)	C18—H18	0.9300
C4—C5	1.351 (4)	C19—C20	1.351 (5)
C4—H4	0.9300	C19—H19	0.9300
C5—H5	0.9300	C20—C21	1.383 (5)
C6—H6A	0.9600	C20—C23	1.519 (4)
C6—H6B	0.9600	C21—C22	1.373 (4)
C6—H6C	0.9600	C21—H21	0.9300
C7—C8	1.315 (3)	C22—H22	0.9300
C7—H7	0.9300	C23—H23A	0.9600
C8—C9	1.465 (3)	C23—H23B	0.9600
C8—H8	0.9300	C23—H23C	0.9600
			
O5—S—O4	116.5 (2)	C10—C11—H11	119.4
O5—S—O3	110.9 (2)	O1—C12—C11	123.1 (2)
O4—S—O3	110.04 (15)	O1—C12—C13	117.6 (2)
O5—S—C17	107.1 (1)	C11—C12—C13	119.3 (2)
O4—S—C17	107.4 (1)	O2—C13—C14	125.4 (2)
O3—S—C17	104.1 (1)	O2—C13—C12	115.2 (2)
C12—O1—H1O	109.5	C14—C13—C12	119.3 (2)
C13—O2—C15	116.5 (2)	C13—C14—C9	120.9 (2)
H6OA—O6—H6OB	92 (1)	C13—C14—H14	119.6
C5—N—C1	120.1 (2)	C9—C14—H14	119.6
C5—N—C6	119.6 (2)	O2—C15—C16	107.3 (2)
C1—N—C6	120.2 (2)	O2—C15—H15A	110.3
N—C1—C2	120.7 (2)	C16—C15—H15A	110.3
N—C1—H1	119.6	O2—C15—H15B	110.3
C2—C1—H1	119.6	C16—C15—H15B	110.3
C1—C2—C3	120.6 (2)	H15A—C15—H15B	108.5
C1—C2—H2	119.7	C15—C16—H16A	109.5
C3—C2—H2	119.7	C15—C16—H16B	109.5
C4—C3—C2	116.4 (2)	H16A—C16—H16B	109.5
C4—C3—C7	119.2 (2)	C15—C16—H16C	109.5
C2—C3—C7	124.5 (2)	H16A—C16—H16C	109.5
C5—C4—C3	121.0 (2)	H16B—C16—H16C	109.5
C5—C4—H4	119.5	C18—C17—C22	119.4 (2)
C3—C4—H4	119.5	C18—C17—S	120.46 (18)
N—C5—C4	121.1 (2)	C22—C17—S	120.07 (19)
N—C5—H5	119.5	C17—C18—C19	119.6 (3)
C4—C5—H5	119.5	C17—C18—H18	120.2
N—C6—H6A	109.5	C19—C18—H18	120.2
N—C6—H6B	109.5	C20—C19—C18	121.8 (3)
H6A—C6—H6B	109.5	C20—C19—H19	119.1
N—C6—H6C	109.5	C18—C19—H19	119.1
H6A—C6—H6C	109.5	C19—C20—C21	118.3 (2)
H6B—C6—H6C	109.5	C19—C20—C23	121.1 (4)
C8—C7—C3	123.7 (2)	C21—C20—C23	120.6 (3)
C8—C7—H7	118.2	C22—C21—C20	121.0 (3)
C3—C7—H7	118.2	C22—C21—H21	119.5
C7—C8—C9	127.7 (2)	C20—C21—H21	119.5
C7—C8—H8	116.1	C21—C22—C17	119.9 (3)
C9—C8—H8	116.1	C21—C22—H22	120.1
C10—C9—C14	118.7 (2)	C17—C22—H22	120.1
C10—C9—C8	118.1 (2)	C20—C23—H23A	109.5
C14—C9—C8	123.1 (2)	C20—C23—H23B	109.5
C9—C10—C11	120.6 (2)	H23A—C23—H23B	109.5
C9—C10—H10	119.7	C20—C23—H23C	109.5
C11—C10—H10	119.7	H23A—C23—H23C	109.5
C12—C11—C10	121.1 (2)	H23B—C23—H23C	109.5
C12—C11—H11	119.4		
			
C5—N—C1—C2	0.5 (3)	O1—C12—C13—C14	178.16 (19)
C6—N—C1—C2	−177.3 (2)	C11—C12—C13—C14	−1.6 (3)
N—C1—C2—C3	0.5 (3)	O2—C13—C14—C9	−179.4 (2)
C1—C2—C3—C4	−1.1 (3)	C12—C13—C14—C9	1.2 (3)
C1—C2—C3—C7	179.0 (2)	C10—C9—C14—C13	0.3 (4)
C2—C3—C4—C5	0.6 (3)	C8—C9—C14—C13	−179.2 (2)
C7—C3—C4—C5	−179.4 (2)	C13—O2—C15—C16	−178.7 (3)
C1—N—C5—C4	−0.9 (3)	O5—S—C17—C18	−19.3 (2)
C6—N—C5—C4	176.9 (2)	O4—S—C17—C18	106.6 (2)
C3—C4—C5—N	0.3 (4)	O3—S—C17—C18	−136.8 (2)
C4—C3—C7—C8	175.4 (2)	O5—S—C17—C22	158.9 (2)
C2—C3—C7—C8	−4.7 (4)	O4—S—C17—C22	−75.3 (2)
C3—C7—C8—C9	−179.7 (2)	O3—S—C17—C22	41.4 (2)
C7—C8—C9—C10	−175.9 (2)	C22—C17—C18—C19	−0.6 (4)
C7—C8—C9—C14	3.5 (4)	S—C17—C18—C19	177.6 (2)
C14—C9—C10—C11	−1.4 (4)	C17—C18—C19—C20	−0.2 (4)
C8—C9—C10—C11	178.0 (2)	C18—C19—C20—C21	1.2 (4)
C9—C10—C11—C12	1.1 (4)	C18—C19—C20—C23	−177.1 (3)
C10—C11—C12—O1	−179.3 (2)	C19—C20—C21—C22	−1.3 (4)
C10—C11—C12—C13	0.4 (4)	C23—C20—C21—C22	176.9 (3)
C15—O2—C13—C14	1.6 (4)	C20—C21—C22—C17	0.6 (4)
C15—O2—C13—C12	−179.0 (2)	C18—C17—C22—C21	0.4 (4)
O1—C12—C13—O2	−1.3 (3)	S—C17—C22—C21	−177.75 (18)
C11—C12—C13—O2	179.0 (2)		

### Hydrogen-bond geometry (Å, °)

**Table:**

*D*—H···*A*	*D*—H	H···*A*	*D*···*A*	*D*—H···*A*
O1—H1O···O6^i^	0.82	1.82	2.632 (3)	170
O6—H6OB···O3	0.85 (1)	2.36 (2)	2.692 (3)	104 (2)
O6—H6OA···O5^ii^	0.86 (3)	1.98 (3)	2.832 (4)	169 (3)
C4—H4···O4	0.93	2.53	3.426 (3)	161
C5—H5···O1^iii^	0.93	2.59	3.217 (3)	125

Symmetry codes: (i) *x*+1/2, −*y*+1/2, *z*−1/2; (ii) −*x*+1, −*y*+1, −*z*+1; (iii) *x*−1/2, −*y*+1/2, *z*+1/2.
